# Improving a fish intestinal barrier model by combining two rainbow trout cell lines: epithelial RTgutGC and fibroblastic RTgutF

**DOI:** 10.1007/s10616-019-00327-0

**Published:** 2019-06-29

**Authors:** Carolin Drieschner, Nguyen T. K. Vo, Hannah Schug, Michael Burkard, Niels C. Bols, Philippe Renaud, Kristin Schirmer

**Affiliations:** 1 Department of Environmental Toxicology, Eawag (Swiss Federal Institute of Aquatic Science and Technology), Dübendorf, Switzerland; 2 Microsystems Laboratory 4, School of Engineering, EPFL (École Polytechnique Fédérale de Lausanne), Lausanne, Switzerland; 3 Department of Biology, McMaster University, Hamilton, ON Canada; 4 Department of Biology, University of Waterloo, Waterloo, ON Canada; 5 Laboratory of Environmental Toxicology, School of Architecture, Civil and Environmental Engineering, EPFL (École Polytechnique Fédérale de Lausanne), Lausanne, Switzerland; 6 Department of Environmental Systems Science, ETHZ (Swiss Federal Institute of Technology in Zurich), Zurich, Switzerland

**Keywords:** Fish-gut-on-chip, Rainbow trout (*Oncorhynchus mykiss*), Epithelial barrier model, Anodized aluminum, Impedance spectroscopy, TEER

## Abstract

An in vitro model of the fish intestine is of interest for research and application in diverse fields such as fish physiology, aquaculture and chemical risk assessment. The recently developed epithelial barrier model of the fish intestine relies on the RTgutGC cell line from rainbow trout (*Oncorhynchus mykiss*), cultured in inserts on permeable membranes. Our aim was to extend the current system by introducing intestinal fibroblasts as supportive layer in order to reconstruct the epithelial–mesenchymal interface as found in vivo. We therefore initiated and characterized the first fibroblast cell line from the intestine of rainbow trout, which has been termed RTgutF. Co-culture studies of RTgutGC and RTgutF were performed on commercially available electric cell substrate for impedance sensing (ECIS) and on newly developed ultrathin, highly porous alumina membranes to imitate the cellular interaction with the basement membrane. Cellular events were examined with non-invasive impedance spectroscopy to distinguish between barrier tightness and cell density in the ECIS system and to determine transepithelial electrical resistance for cells cultured on the alumina membranes. We highlight the relevance of the piscine intestinal fibroblasts for an advanced intestinal barrier model, particularly on ultrathin alumina membranes. These membranes enable rapid crosstalk of cells cultured on opposite sides, which led to increased barrier tightening in the fish cell line-based epithelial–mesenchymal model.

**Electronic supplementary material:**

The online version of this article (10.1007/s10616-019-00327-0) contains supplementary material, which is available to authorized users.

## Introduction

Rainbow trout (*Oncorhynchus mykiss*) cell lines have been established from a variety of different tissues and organs and are indispensable for mechanistic investigations in fish based research (Bols and Lee 1991; Castano et al. 2003; Bols et al. 2005, 2017). Great success was achieved in the past years in demonstrating the potential of fish cell lines as alternatives for the replacement of animal testing (Schirmer 2006; Tanneberger et al. 2012). The attractiveness of rainbow trout cell lines as economic and easy to handle source to obtain reliable and reproducible results is further increasing with the development of functional tissue analogues (Malhão et al. 2013; Geppert et al. 2016; Minghetti et al. 2017), technical innovations in label free bio-sensing (Brennan et al. 2012; Curtis et al. 2013; Widder et al. 2015; Tan and Schirmer 2017) and computational modelling (Stadnicka et al. 2012; Stadnicka-Michalak et al. 2014, 2015).

Several in vitro approaches mimicking the fish intestine have been performed because in the aquatics sciences this organ is of manifold interest, such as in fish physiology, aquaculture and environmental risk assessment. The intestinal tract function includes the uptake of nutrients from the diet, in blocking the entry of pathogens and hazardous chemicals, in regulating ion homeostasis and in being a large part of the immune system (Grosell et al. 2011). The current in vitro model of the fish intestine is based on the epithelial-like cell line RTgutGC (Kawano et al. 2011). The cells are cultured in commercial transwell inserts on a permeable membrane and form a barrier between two compartments, representing the intestinal lumen on one side and the interior of the organism on the other (Geppert et al. 2016; Minghetti et al. 2017). Recently, we developed ultrathin alumina membranes as a novel scaffold for RTgutGC cells. Compared to insert membranes, this substrate is at least 10 times thinner, more porous and thus also more permeable and can be considered as a close analogue to the delicate basement membrane that underlines the epithelial cells in vivo (Drieschner et al. 2017). Optimal mimicry of basic intestinal architecture calls for further refinements by e.g. having additional intestinal cell types that help to improve the intestinal barrier function and allow for a broader application of the model.

Intestinal fibroblasts are one cell type of interest as an addition to in vitro models of the intestine. In vivo, the intestinal epithelium is underlined by fibroblasts as supportive layer (Powell et al. 2011). Fibroblasts originate from the mesenchyme and have diverse functions in the organism, including production of extracellular matrix, immune stimulation and wound healing (Sorrell and Caplan 2009; Ingerslev et al. 2010). The crosstalk between epithelial cells and fibroblasts is expected to be essential for epithelial barrier development and functionality (Yasugi 1993; Simon-Assmann et al. 2007; Shaker and Rubin 2010). Lately, 2D and 3D in vitro models of the human intestine have been developed with the aim to remodel the epithelial–mesenchymal interface. These examinations report improved wound healing (Seltana et al. 2010), induced differentiation and proliferation of epithelial cells (Visco et al. 2009), changed sensitivity patterns towards drug exposure (Hoffmann et al. 2015) and more in vivo like transepithelial electrical resistance (TEER) values (Pereira et al. 2015). However, to date, no fish intestinal fibroblast cell line has been described.

Thus, to initiate further development of an increasingly realistic in vitro equivalent of the fish intestine, we developed and characterized the first fibroblast-like cell line from the intestine of rainbow trout. Furthermore, we established co-cultures with the epithelial-like cell line RTgutGC on commercial, planar electrode containing solid supports (electric cell substrate for impedance sensing; ECIS) and on the recently developed ultrathin, highly porous alumina membranes. Cellular resistance, measured by impedance spectroscopy, was used to determine whether fibroblasts have positive effects on the barrier tightness in epithelial–mesenchymal co-cultures.

## Materials and methods

### Cell culture

The rainbow trout intestinal epithelial cell line, RTgutGC (Kawano et al. 2011), was routinely cultured in Leibovitz’s L-15 medium (Invitrogen, Switzerland), supplemented with 5% fetal bovine serum (FBS; PAA, Switzerland) and 1% gentamycin (GIBCO, Invitrogen, Switzerland) at 19 ± 1 °C under normal atmosphere in the dark. The initiation and characterization of the intestinal fibroblast cell line, RTgutF, is described in detail in the following.

### Establishment of the RTgutF cell line

#### Initiation

The RTgutF cell line was developed from tissue fragment cultures of the anterior intestine of a sexually mature male rainbow trout with an approximate weight of 1150 g in Dr. Bols’ laboratory in the Department of Biology at the University of Waterloo (Waterloo, ON, Canada). After euthanizing the fish with an overdose of the anaesthetic MS222 (Syndel, Canada), the anterior part of the intestinal tract was removed and placed in a petri dish. The exterior surface of the excised tissue was gently rinsed with the rinsing solution: Mg^2+^ and Ca^2+^-free Dulbecco’s phosphate-buffered saline (DPBS; Lonza, Canada) with 300 U/mL penicillin and 300 μg/mL streptomycin. A bulb transfer pipette filled with the rinsing solution was inserted into one open end of the intestinal tube to flush the lumen content; this cycle was repeated ten times. Then, the gut was cut open longitudinally and rinsed with the rinsing solution for five more times, as previously done to establish the RTgutGC cell line (Kawano et al. 2011). A scalpel blade was used to scrape off the internal intestinal folds to mechanically remove as much epithelial cell mass as possible. The tissue was then immersed sequentially in several petri dishes filled with the rinsing solution to clean the tissue. The resulting tissue was minced into small pieces of about 1 mm^2^. The tissue fragments were rinsed again for three more times and then placed in 25 cm^2^ tissue culture flasks (Falcon, Canada), with five fragments per flask, in L-15 medium with 10% FBS (Sigma Aldrich, Canada), 200 U/mL penicillin, and 200 μg/mL streptomycin at 19 °C ± 1 °C under normal atmosphere in the dark.

#### Propagation

Medium was renewed daily during the first week and then every 3 days afterwards during the two following weeks. Tissue fragments were kept in the flasks until there were enough adherent cells outgrowing from the explants to carry out the first passage. Primary cultures that had cells with an epithelial-like morphology were discarded. The original flask that gave rise to the RTgutF cell line had extensive networks of fibroblastic cells outgrowing from the explanted gut tissues. The fibroblastic cells were sub-cultured with trypsin/EDTA solution (Lonza, Canada) for the first time after 2 months in culture. The passaged cells were transferred into a new cell culture vessel and kept in L-15 medium with 15% FBS and 1% gentamycin at 19 ± 1 °C under normal atmosphere in the dark, to expand the cell progeny population. For routine culture, the medium was renewed once per week and cells were split every one to 2 weeks, when reaching confluency of 80–90%, by rinsing with trypsin/EDTA (Biowest, France) solution and splitting in a 1:2 or 1:3 ratio in 75 cm^2^ cell culture flasks (TPP, Switzerland). Permanent cultures of RTgutF were sent to Dr. Schirmer’s laboratory in the Department of Environmental Toxicology at Eawag, the Swiss Federal Institute of Aquatic Science and Technology (Dübendorf, Switzerland), where further cellular characteristics and functional properties of the cell line were studied.

#### Cryopreservation

RTgutF cell cultures have been successfully cryopreserved in L-15 supplemented with 15% FBS and 10% (v/v) dimethyl sulphoxide (DMSO; Sigma Aldrich, Switzerland) in liquid nitrogen. Success was judged by a recovery rate of 70–80% of the cell cultures at different passages (13, 26, 52) upon thawing.

#### Cell line origin—species identification

DNA barcoding for the cytochrome c oxidase subunit 1 (COX1) was performed at the Biodiversity Institute of Ontario (Guelph, Canada) to authenticate RTgutF cell line as derived from *O. mykiss*. A universal PCR primer cocktail designed for teleosts along with all the PCR thermocycling conditions were performed as described by Ivanova et al. (2007) to amplify a 652-bp region of the rainbow trout COX1 gene in RTgutF. The amplicon was sequenced and matched to the species identification in both Barcode of Life Data (BOLD) (Ratnasingham and Hebert 2007) (http://www.barcodinglife.org) as well as the NCBI BLAST databases (http://www.ncbi.nlm.nih.gov/ BLAST).

#### Growth characteristics

For the proliferation assay, RTgutF cells at passage 22–25 were seeded at a density of 15,000 cells/cm^2^ in L-15 medium supplemented with 15% FBS and 1% gentamycin in 12-well plates (Greiner-Bio-One, Switzerland). Plates were incubated for 5 h at 19 ± 1 °C in the dark to assure cell attachment. Thereafter, medium was aspirated and replaced with 2 mL of L-15 containing 0, 5, 10 or 20% of FBS and the antibiotics. The cell number was determined at day 1, 3, 7, 10, 14 and 17 after seeding.

On the respective day, three wells per FBS concentration were washed twice with Versene (Gibco, Switzerland) followed by trypsinization. Cells were resuspended in the trypsin solution and the reaction was stopped by adding L-15 containing 15% FBS. The cell number was determined using a Casy TTC cell counter (Schärfe System GmbH, Germany). The doubling time was determined by fitting an exponential growth equation (Eq. 1) and calculations using the rate constant k (Eq. 2), which is expressed in reciprocal of the x-axis time units.

1 $$Cell\; number = Cell \;number_{t0} *e^{{\left( {k*time} \right)}}$$

2 $$Doubling\; time = \frac{\ln \left( 2 \right)}{k}$$

#### Telomerase activity

As an indicator of cell culture longevity, telomerase activity was assessed using Telo TAGGG Telomerase PCR ELISA (Roche, Germany) following manufacturer’s protocols. In brief, RTgutF cells (20,000 cells) were harvested at different passages, lysed and telomeric repeat amplification was performed with provided substrate primers (20 min elongation, 5 min inactivation and 30× amplification cycles). Products were denatured and hybridised with Digoxigenin (DIG), followed by immobilisation with biotin and streptavidin coating. Samples were semi-quantitatively assessed using the internal standard and horseradish peroxidase (Anti-DIG-HRP). The limit of detection was considered as the two-fold background activity. Protein content was assessed by bicinchoninic acid protein assay kit (Pierce, USA) following the instructions of the manufacturer. Relative telomerase activity was normalized to 1 mg/mL total protein.

### RTgutF and RTgutGC cell cultures on solid support with integrated electrodes

RTgutF and RTgutGC cells were cultured either as mono- or as co-cultures directly on electric cell substrate for impedance sensing (ECIS), specifically on 8-well chips, each with 20 inter-digitated finger electrodes (8W20idf PET, Applied BioPhysics, ibidi, Germany) to measure the electrical properties of approximately 4000–8000 cells. For monitoring growth kinetics, RTgutF or RTgutGC cells were seeded at a density of 25,000 cells/cm^2^ each. For monitoring barrier formation, cells were seeded at a concentration of 55,000 cells/cm^2^ each. For co-culture initiation, RTgutF seeding was performed 3 days prior to RTgutGC seeding. During experimentation, the medium containing 5% FBS for all applications was fully changed every one to 2 days.

### RTgutF and RTgutGC cell cultures on ultrathin permeable alumina membranes

Ultrathin alumina membranes were fabricated, prepared and used as previously described (Drieschner et al. 2017). According to the fabrication process, the membranes are realized within a silicon frame for support, the entire unit will be referred as alumina chip. Alumina chips feature a flat side (top) and a micro-structured side (bottom) with a microwell array to access the nanoporous membrane. Prior to cell culture, alumina chips were sterilized in 70% ethanol for 20 min and membranes were coated with 50 µg/µL fibronectin (Roche, Germany) in distilled autoclaved water for 2 h on each membrane side and placed in L-15 supplemented with 5% FBS for 1 day before cell seeding. For monocultures, RTgutGC or RTgutF cells were seeded on the top side of the membrane, each with a density of 55,000 cells/cm^2^. Co-cultures were established in two ways: (1) RTgutGC cells were seeded on top of RTgutF cells and (2) RTgutF and RTgutGC cells were seeded on opposite sides of the membrane. For (1) RTgutF cells were pre-cultured on the top side of the membrane for 3 days, followed by seeding of RTgutGC, each at a density of 55,000 cells/cm^2^. For (2) RTgutF cells were pre-cultured on the bottom side for 3 days, followed by seeding of RTgutGC on the top side, each with a seeding density of 55,000 cells/cm^2^. After cell attachment, which occurred within 2 h after seeding, the alumina chips were placed on specifically designed polycarbonate holders (Drieschner et al. 2017) in 24 well plates to allow media supply from both sides of the membrane and media was exchanged every one to 2 days, which contained 5% FBS for all applications.

### Impedance spectroscopy

The electrical properties of the cell sheets were evaluated by non-invasive impedance (Z) spectroscopy and integrates information about cell attachment and cell–cell connections, cell growth and transepithelial electrical resistance (TEER).

Impedance spectroscopy involved two steps: determining the baseline in the absence of cells and determining the signal in the presence of cells. For baseline analysis, both types of chips (commercial ECIS and self-made alumina chips) were first pre-equilibrated in L-15 supplemented with 5% FBS for 1 day. The ECIS chip was then directly connected to the impedance analyser (Gamry Instruments, Germany). The alumina chip was placed in an Endohm-6 chamber (World Precision Instruments, Germany), which was then connected to the impedance analyser. Impedance spectra were recorded from 75 to 300 kHz and an amplitude of 10 mV for ECIS chips and from 6 to 300 kHz and an amplitude of 20 mV for alumina chips. The impedance spectra vary between the two set-ups due to the distinct electrode configurations. Impedance profile and corresponding phase angle were analysed for each system to select optimal frequencies for further analysis as described below.

**Fig. 1:**
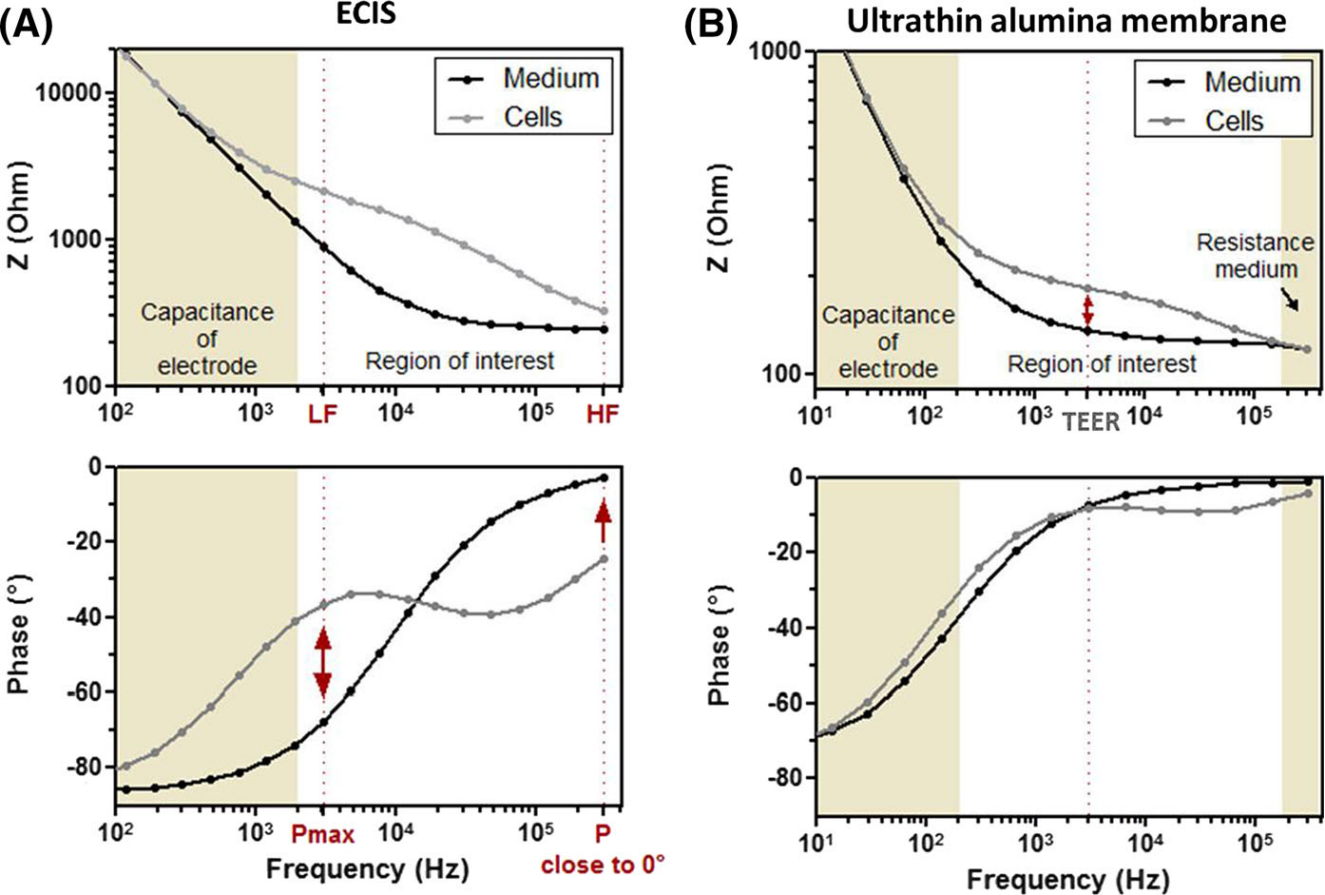
Impedance spectra and corresponding phase angle of a high density cell culture on electric cell substrate for impedance sensing (ECIS) and on ultrathin alumina membranes. Impedance (Z) and phase angle are plotted against the frequency and show typical profiles for cell containing and cell-free **a** ECIS chips and **b** ultrathin alumina membrane chips. Z spectra are mainly dominated by the electrode capacitance in the low frequency range, while the region of interest provides information about the electrical properties of the cells. For ultrathin alumina membranes the Z spectrum is additionally characterized by the dominating resistance of the medium at the higher frequency range. The phase informs about the resistive (→ 0°) or capacitive (− 90°) character of the system and can be used to determine a low frequency (LF) and a high frequency (HF) within the ECIS system by selecting the maximal phase angle difference (P_max_, before curves are crossing) and the phase indicating a resistive character of the system (P close to 0°). For alumina membranes the phase can be used to determine the optimal frequency for transepithelial electrical resistance (TEER) measurements, which normally correlates with the crossing phase angle curves

For ECIS chips, cells are directly cultured on the interdigitated finger electrodes, which provides very high measure sensitivity. This arrangement allows to adapt the principal of low and high frequency analysis, as characterized and applied by Meissner et al. (2011) and Benson et al. (2013), to distinguish between paracellular and transcellular resistance. In brief, at low frequency (LF), the cell membrane acts as insulator and the ionic current predominantly flows paracellular. Thus, measured resistance values provide information on cell-substrate and cell–cell adhesion. At high frequency (HF), the cell membrane capacitor is short-circuited and resistance of intracellular matter has the main effect on impedance. Figure 1a demonstrates selection criteria for LF and HF. At the lower end of the frequency range, the impedance spectrum is dominated by the capacitance of the electrodes (top graph). Hence, the region of interest starts at around 1 kHz. Further, the phase angle (bottom graph), indicating if a system behaves like a resistor (→ 0°) or capacitor (→ 90°), is used to determine LF and HF. The highest measurement sensitivity for LF is achieved at the maximal phase angle difference (P_max_) within the LF range (< 12 kHz; crossing curves). Thus, a LF of 3 kHz for ECIS was chosen. HF is selected where the phase angle is close to 0° (P close to 0°) and the membrane capacitor is short-circuited (end of spectra). Therefore, we have chosen 300 kHz. It is important to keep in mind that neither LF or HF-based resistance values are purely indicative for paracellular or transcellular events (phase angle profile). Yet, for simplification, we refer to LF obtained values as paracellular resistance and to HF obtained values as transcellular resistance.

Impedance measurement for cells cultured on alumina membranes is less sensitive because counter electrodes are located on opposite membrane sides and resistance of the culture membrane and the media have a dominating effect at the higher frequency range and thus do not allow to retrieve information about the transcellular resistance (Fig. 1b). In this set-up the region of interest is limited by the capacitance of the electrodes at the LF end and by stray capacitance at the HF end (top graph). Resistance values to calculate TEER are obtained at the frequency with the greatest difference of impedance between the presence and absence of cells (van der Helm et al. 2017). In the phase-angle plot (bottom graph) this frequency often correlates with the crossing of curves from cell and blank measurements. This resistance was determined at 3 kHz. For TEER calculation, resistance values were multiplied with the membrane area.

### Microscopy

#### Light microscopy

Phase-contrast images were obtained with cells cultured in 24 well plates (Greiner-bio-one, Switzerland) using a Leica DMI600 inverted microscope.

#### Fluorescence microscopy

Immunocytochemical staining was applied to visualize collagen and vimentin as fibroblast markers, ZO-1 as indication of tight junction formation, and DAPI for staining the cell nuclei. To initiate staining, cells were seeded on coverslips (Thermanox, Thermo Fisher, Switzerland) or four-chamber tissue culture Lab-Tek slides (Nunc, Thermo Fisher, Canada) at a density of 55,000 cells/cm^2^ and left to attach and spread out overnight. In addition, potential multilayer formation of high density fibroblast cultures after a culture period of 14 days was verified through cross-section analysis (z-stack) as described in (Drieschner et al. 2017).

For collagen (type I, alpha 1; COL1A1, antikörper-online.de, Switzerland) immunostaining, cells were fixed with 3.7% paraformaldehyde (Invitrogen, Switzerland) in phosphate buffered saline (PBS) for 10 min at room temperature. After washing in PBS, cells were permeabilized for 15 min with 0.2% Triton X-100 in PBS. After a further washing step with PBS containing 0.1% Triton X-100, cells were incubated in Image-iT (Invitrogen, Switzerland) for 30 min, rinsed with PBS and primary antibody (diluted 1:40 in 0.5% goat serum and 0.05% Triton X-100 in PBS) for COL1A1 was applied overnight at 4 °C. The next day, cells were washed with 0.1% Triton X-100 in PBS and the secondary antibody, Alexa Fluor 488^®^-conjugated goat-anti-rabbit IgG (Invitrogen, Switzerland), was applied at a dilution of 1:1000 for 1 h at room temperature. Samples were washed in 0.1% Triton X-100 in PBS and incubated with 10.9 µM DAPI (Invitrogen, Switzerland) in PBS for 5 min. After repeated washing in 0.1% Triton X-100 in PBS and PBS only, coverslips were mounted on microscope slides using ProLong^®^ Gold antifade reagent (Life Technology, United States). A negative control staining is depicted in supplemental material Figure S3. Imaging and analysis was performed on a Leica SP5 Laser Scanning Confocal Microscope (Leica, Switzerland) using the LAS AF Lite 2014 software.

The vimentin immunostaining followed the same procedure as previously described (Bloch et al. 2016). Briefly, cells were fixed in 100% ice-cold methanol for 15 min at 4 ± 1 °C, followed by a quick wash in PBS to rehydrate cells. Fixed cells were incubated for 1 h in a blocking buffer containing 10% goat serum, 3% bovine serum albumin, and 0.1% Triton X-100 in PBS and then probed with mouse monoclonal anti-vimentin antibody (Sigma Aldrich, Canada) diluted at 1:200 in blocking buffer for 1 h at room temperature. The secondary antibody was Alexa Flour 488^®^-conjugated goat anti-mouse IgG used at 1:1000 dilution in PBS for 1 h. Cells were then washed five times with PBS, allowed to dry and mounted in Fluoroshield medium containing DAPI (Abcam, Canada). A negative control staining is depicted in supplemental material Figure S3. Fluorescence images were taken with a Zeiss LSM510 laser-scanning microscope (Zeiss, Canada) and confocal images were acquired and analyzed using the ZEN lite 2011 software.

#### Electron microscopy

For cross-section images, RTgutF and RTgutGC cells were cultured on opposite sides of alumina membranes for 10 days. Thereafter, cells were washed with PBS and fixed in 2.5% glutardialdehyde for 1 h, followed by postfixation in 2% osmium tetroxide for 1 h and block staining with 2% uranyl acetate for 1 h with washing steps in between. Dehydration was performed in a graded series of 30, 50, 70, 90, and 100% ethanol, followed by twice water-free 100% ethanol, 30 min each. Subsequently, alumina membranes with cells were impregnated, first with 33% resin (EMbed 812, Electron Microscopy Science, USA) and then with 66% resin in water-free ethanol for 1.5 h each. Thereafter, alumina chips with cells were submerged in 100% resin twice for 2 h each. All impregnation steps were performed at room temperature in small plastic dishes. The sample was completely submerged in the resin and then taken out of the resin bath. Excess of resin was allowed to drain in an upright position for another 1.5 h at room temperature. The sample was then transferred to the oven heated up to 60 °C, where polymerization of the resin proceeded for 2 days. The polymerized alumina chip with cells was cooled to room temperature and directly mounted right-side-up onto scanning electron microscopy (SEM) stubs with conductive carbon cement. The specimen was sputter coated with 6 nm platinum. In the focused ion beam (FIB)-SEM (FEI Helios 600i), the sample was screened with an electron beam at high accelerating voltage (30 kV) and imaged in the backscattered electron (BSE)-mode to select a region of interest. The sample was brought to a stage position, where electron beam and ion beam coincide (stage tilt at 52°) and a trench was milled with the FIB at 30 kV and 9.3 nA to open the sample. The resulting cross section was polished at an ion current of 2.5 nA and then transmission electron microscopy images were taken with an electron beam of 2 kV and 0.34 nA. Images were acquired in the BSE-mode at appropriate tilt correction and a dwell time of 30 µs. Neighbouring images were merged to full panorama view.

### Statistical analysis

**Fig. 2:**
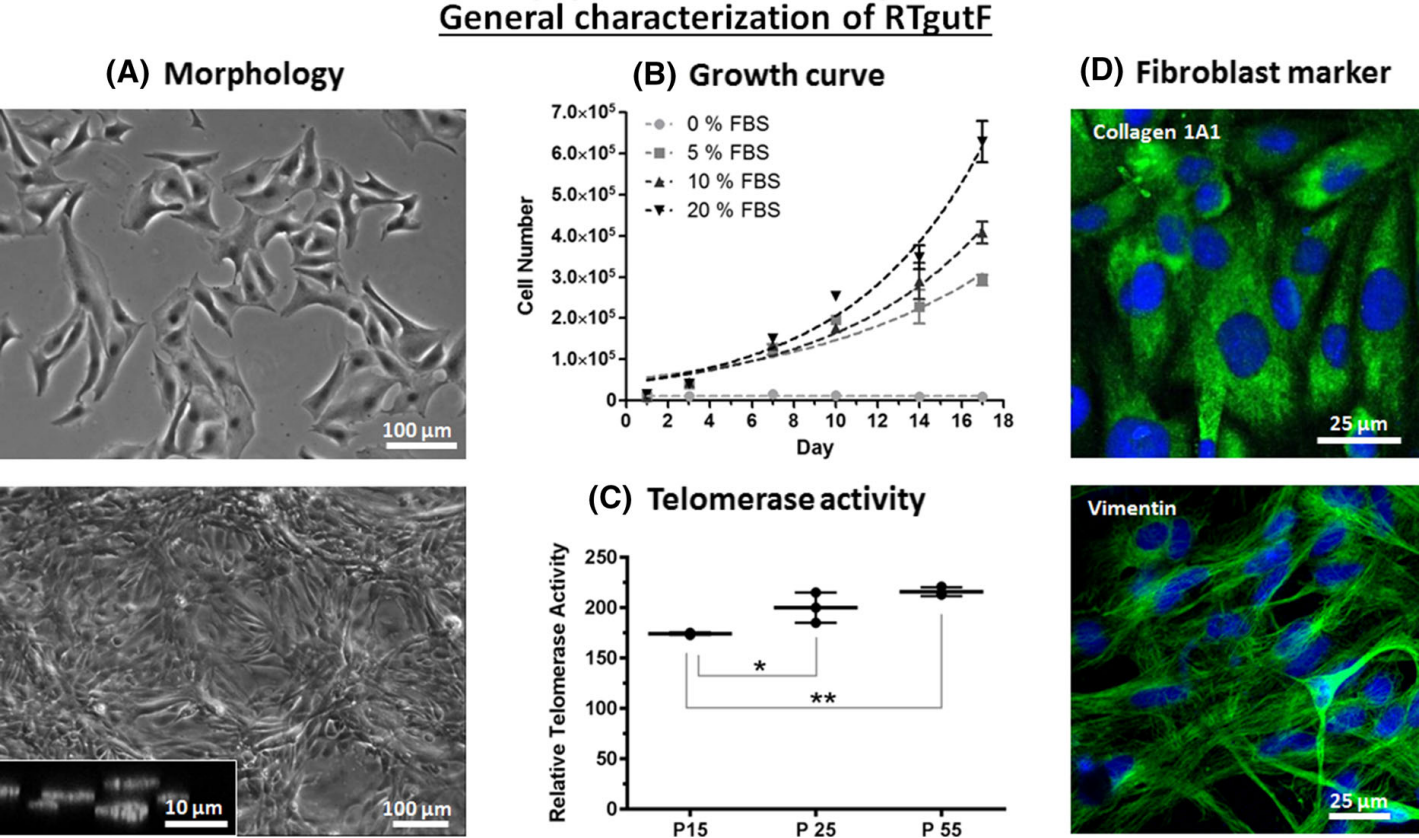
General characterization of RTgutF grown on a conventional plastic tissue culture surface. **a** Morphology as viewed by phase contrast microscopy of cells in low density 1-day old cultures (top) and in high density (bottom) 14-day old cultures. At high densities, RTgutF cells partially formed multiple layers (inlay—cross section of DAPI stained cell nuclei, bottom image). **b** Cell number (mean ± SD; n = 3) in cultures over time in L15 with different FBS concentrations. **c** Relative telomerase activity (mean ± SD; n = 3) at different passages. The statistical differences are denoted: *p* < 0.05 by * and *p* < 0.01 by **. **d** Immunocytochemical staining and confocal imaging demonstrate collagen 1A1 (top image, green staining) and vimentin (bottom image, green staining) in RTgutF. Cell nuclei were counterstained with DAPI (blue). (Color figure online)

**Fig. 3:**
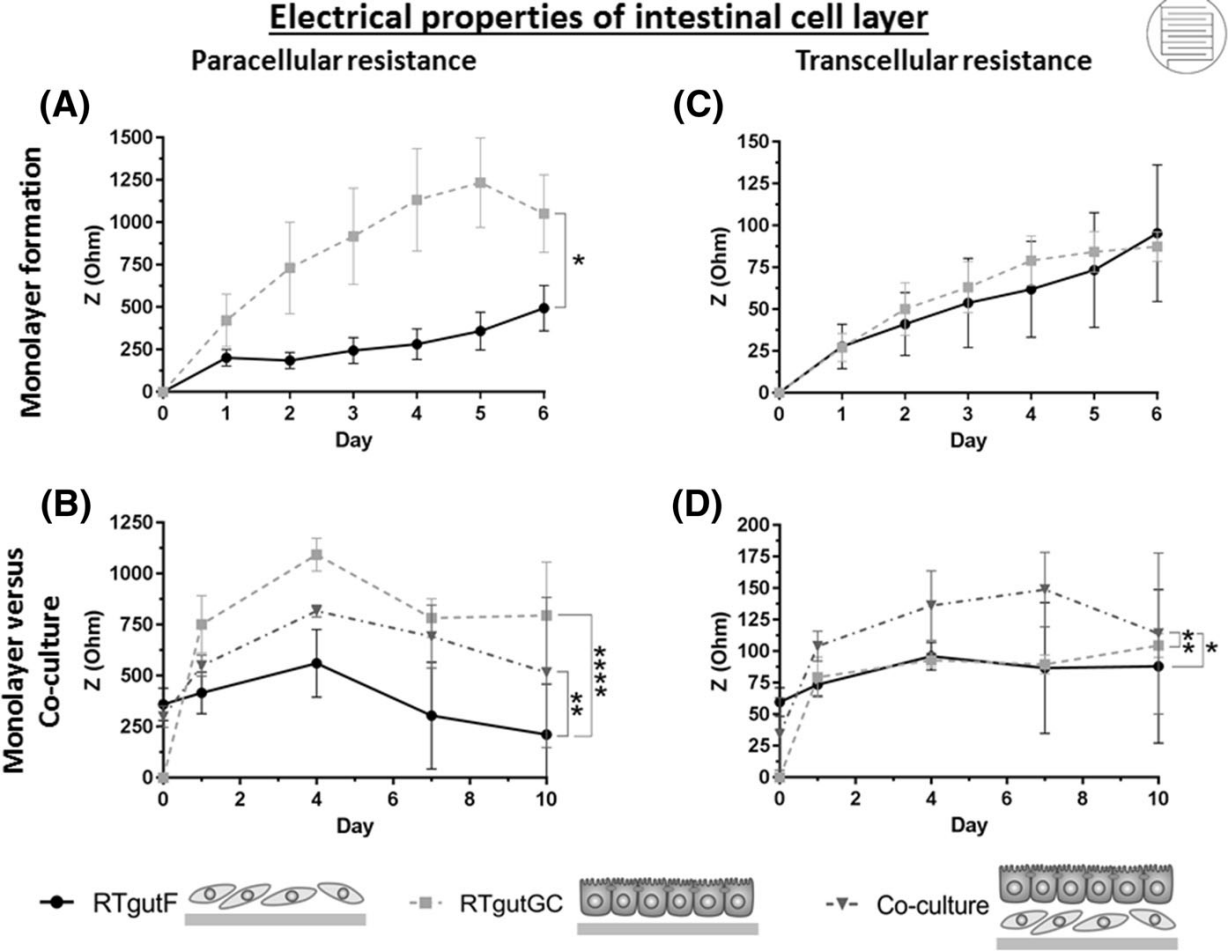
Development of paracellular and transcellular resistance in cultures of RTgutF and RTgutGC alone or together on electric cell substrate for impedance sensing (ECIS). Three types of culture organizations were initiated on solid plastic supports with integrated electrodes (ECIS system) as shown in the schematics at the bottom of the figure. Paracellular resistance (**a**, **b**) was determined at a low frequency and reflects cell adhesion and formation of cell–cell contacts. Transcellular resistance (**c**, **d**) was measured at high frequency and represents cell density in this specific case. In (**a, c)**, RTgutF and RTgutGC were seeded at a moderate density (25,000 cells/cm^2^; confluency 50%), to monitor resistance changes during the formation of a confluent monolayer over a time period of 6 days. In (**b**, **d)**, RTgutF and RTgutGC were seeded at a high density (55,000 cells/cm^2^; confluency 100%) each, when cultured alone or in combination for 10 days, to compare the resistances of the individual monolayers to the co-culture. Data represent the mean ± SD; n = 4. The statistical differences are denoted: *p* < 0.05 by *, *p* < 0.01 by ** and *p* < 0.0001 by ****

**Fig. 4:**
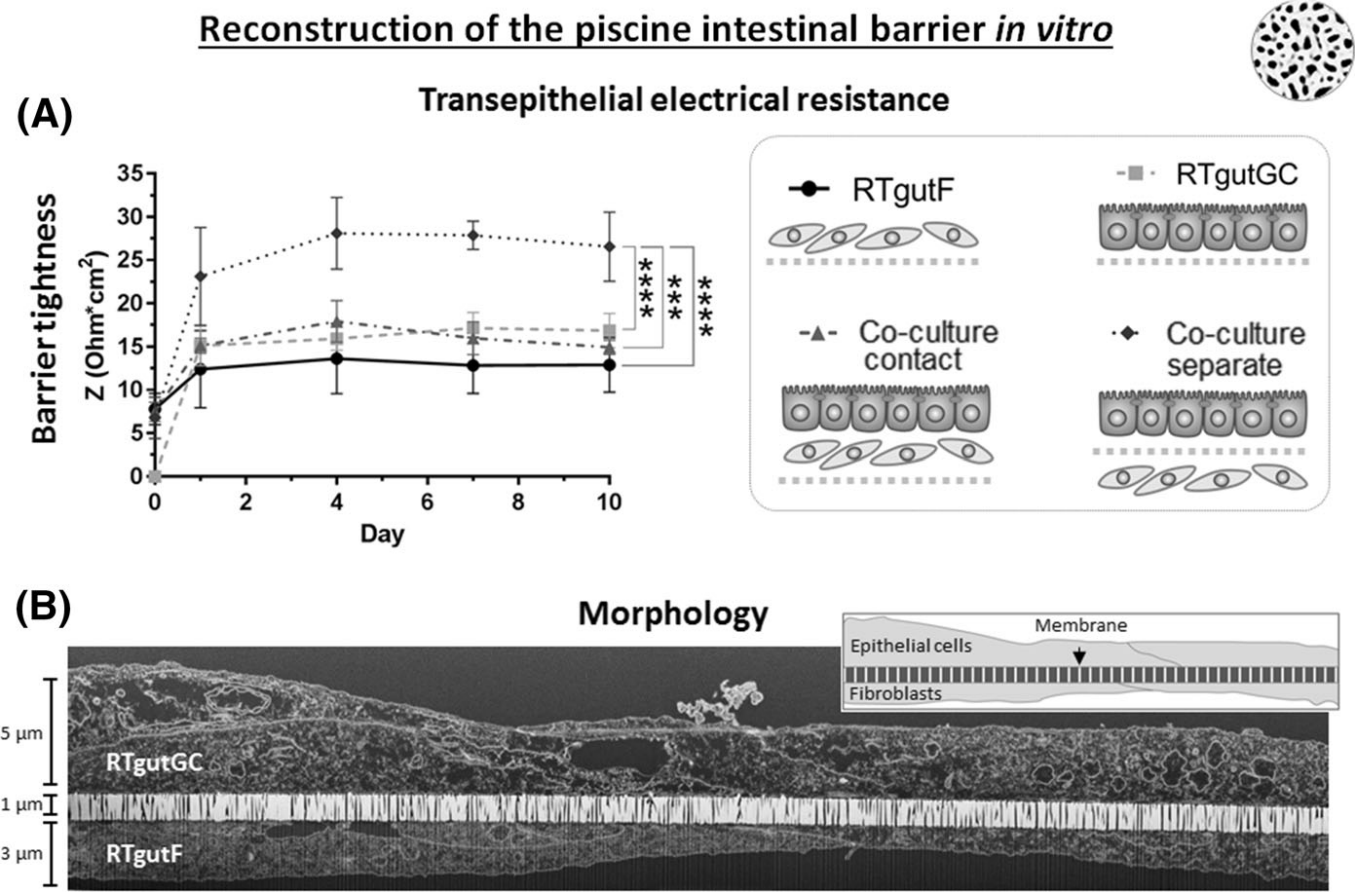
Culture of RTgutGC and RTgutF alone or together on ultrathin alumina membranes. Four types of culture organizations were initiated on ultrathin alumina membranes as shown on the right in (**a)** and the development over 10 days of transepithelial electrical resistance (TEER) as measured by impedance is graphed on the left in (**a)**. RTgutF and RTgutGC were seeded either individually or as contact co-culture at a high density (55,000 cells/cm^2^; confluency 100%) each on the top side of the membrane (depicted as grey dotted line in **a**, right side). For separate co-culture initiation RTgutF and RTgutGC were seeded at an identical seeding density on opposite membrane sides (grey dotted line separating the two cell layer). Data represent the mean ± SD; n = 4. The statistical differences are denoted: *p* < 0.001 by *** and *p* < 0.0001 by ****. **b** Cross section of a co-culture where cells are separated via membrane (10 days). The sample was prepared with focused-ion-beam and imaged with transmission electron microscopy. The inlay is a simplified drawing of the cross section

Results were represented as mean ± standard deviation (SD) with n indicating the number of independently conducted experiments. Statistical analysis was performed using Graphpad Prism^®^ software (Prism 7.04 for Windows) for (1) comparing relative telomerase activity at different passages by one-way ANOVA, together with Tukey’s post hoc test (Fig. 2c); (2) comparing the resistance of RTgutGC versus RTgutF during monolayer formation by an unpaired *t* test (Fig. 3a, c); (3) comparing the resistance of RTgutGC monolayer versus RTgutF monolayer versus Co-culture by two-way ANOVA, together with Tukey’s post hoc test (Figs. 3b, d and 4a). The level of significance was set at probabilities of *p < 0.05, **p < 0.01, ***p < 0.001 and ****p < 0.0001.

## Results and discussion

Focusing on the recreation of basic intestinal architecture, the aim of this study was to combine absorptive epithelial cells, which face the intestinal lumen in vivo, and supportive fibroblasts, which are the main cell type in the underlying connective tissue. In order to achieve this, we followed three lines of investigation. First, a novel fibroblast cell line from rainbow trout, RTgutF, was initiated and characterized. Second, this cell line was used alone or in combination with the intestinal epithelial cell line RTgutGC on solid, non-porous supports to evaluate their electrical properties. Third, ultrathin nanoporous alumina membranes were used as basement membrane analogue to allow separation of the two cell types on one hand, while still promoting cellular cross-talk on the other.

### The RTgutF cell line

Based on the explant outgrowth technique, which was previously applied for the establishment of cell lines from various organs and tissues of different teleost species (Kawano et al. 2011; Vo et al. 2015; Pham et al. 2017), we succeeded in establishing the first intestinal fibroblast cell line from rainbow trout. Cells have been passaged more than 60 times and been cryopreserved successively.

Primary cultures initially generated fibroblastic and epithelial cell populations; however, fibroblastic cells were successfully separated. Early passages (< 6) of RTgutF cells showed heterogeneous cell morphology, with a majority of thin elongated cells forming a network like structure and a minority of plump outspread cells (see supplemental material, Figure S1). From passage 6 onwards, RTgutF cell populations changed to a uniform morphology. At low densities cell bodies are spread out (Fig. 2a top) while at high densities the cell shape becomes elongated with centred nuclei. If left to grow upon confluency, cells start to form multiple cell layers (Fig. 2a bottom—inset) and detach within 14–21 days after reaching confluency. In contrast, RTgutGC cells remain in monolayer for up to several month after reaching confluency. This reflects a specific behaviour of the individual cell types, and is coherent with the fact that close contact among in vitro normal epithelial cells leads to contact inhibition and cell cycle arrest (Puliafito et al. 2012). Fibroblasts, on the other hand, are known to freely move within the 3D arrangement of connective tissue and are thus less strongly adherent to their culture surface in vitro (Hay 1995).

RTgutF cells respond to increased concentrations of serum by an acceleration of proliferation (Fig. 2b). Doubling times calculated from the growth curves ranged from 6½ days for 5% to 5 days for 10%, to 4½ days for 20% FBS. In contrast, in the absence of serum, no cell proliferation occurred; yet, RTgutF cells remained attached to the culture surface. For routine cell culture, a reduced FBS concentration of 10% can be recommended. This concentration supports normal fibroblast morphology and optimal proliferation rates, which are comparable to the proliferation profile of RTgutGC cells (Kawano et al. 2011).

The RTgutF cell line appears to be infinite based on the number of passages achieved today. In support of this observation, cells express slightly rising levels of telomerase over passages (Fig. 2c), an enzyme known to extend cell longevity by extending the chromosome termini, which are otherwise shortened with each cell cycle (Dey and Chakrabarti 2018). In some species, including rainbow trout, telomerase activity is not limited to stem cells, but was detected in all somatic cells (Klapper et al. 1998). Indeed, telomerase activity in RTgutF cells was comparable to that of other normal fish cell lines and one telomerase transfected mammalian cell line (see supplemental material, Figure S2).

Further evidence that supports the notion that RTgutF cells are fibroblasts was provided by immunocytochemical staining of collagen 1 (Col1A1) and vimentin. Both proteins are abundantly expressed throughout the cell body of RTgutF cells (Fig. 2d). In mammalian cell culture, these proteins are frequently used markers of fibroblasts (Franke et al. 1982; Burkard et al. 2015). In rainbow trout, no finite biochemical markers are defined yet for cell type identification (Bols et al. 2005). However, fibroblast specific expression of collagen 1 has been verified for rainbow trout dermal skin cell cultures and tissue samples (Rakers et al. 2011), which supports the potential use of collagen 1 expression as marker for rainbow trout cell line classification. Vimentin expression in rainbow trout is less clear and expression patterns have been described as significantly different from mammals (Herrmann et al. 1996). Thus, the information of positive vimentin expression in RTgutF cells should be rather seen as an enrichment of the literature of the notoriously complex expression patterns of vimentin in fish than as a definite fibroblast marker.

RTgutF species identity was verified by PCR amplification of RTgutF DNA of the 652 base pair long COX1 fragment that yielded a 100% sequence identity match to reference sequence profiles derived from *O. mykiss* (see supplemental material, Figure S4).

With the establishment of RTgutF, the first intestinal fibroblast-like cell line from rainbow trout, it was possible to extend the current RTgutGC model. The supportive function of fibroblasts was analyzed by co-culture initiation on solid substrate and on porous supports, respectively.

### Impedimetric characterization of intestinal cells on solid support

In a first approach, epithelial RTgutGC and fibroblastic RTgutF cells were cultured on electric cell substrate for impedance sensing (ECIS), which provides a solid, non-porous cell culture interface and allows evaluation of electrical properties of cells. These were examined by non-invasive impedance measurements and permitted to evaluate paracellular resistance at low frequency, which is indicative of cell connections and cell adhesion, and transcellular resistance at high frequency, providing information on cell viability.

In the process of forming a confluent monolayer, RTgutF and RTgutGC cells revealed starkly different paracellular resistance profiles (Fig. 3a). RTgutGC presented a gradual increase in resistance values, which was at all times greater compared to RTgutF, with the maximum difference in resistance being about four-fold. Distinctly different profiles were also observed for RTgutF and RTgutGC cells seeded as densely packed monolayer with a confluency of 100% (Fig. 3b). Here, the paracellular resistance of RTgutGC was about two-fold higher than of RTgutF, which indicates stronger cell–cell and cell-substrate connections of RTgutGC and is typical for epithelial cells (Hay 1995). Indeed, the formation of tight junctions between adjacent cells has a strong effect on the paracellular resistance profile (Benson et al. 2013) and was previously verified for RTgutGC by formation of a strong and continuous line of stained ZO-1, a protein of the tight junction complex, on the apical cell periphery (Geppert et al. 2016; Drieschner et al. 2017; Minghetti et al. 2017). In comparison, RTgutF exhibit a weaker and discontinuous line of ZO-1 at the cell-to-cell boundaries (see supplemental material, Figure S5), which is typical for movable fibroblasts (Sorrell and Caplan 2009). For co-culture initiation, RTgutGC was seeded directly on top of RTgutF. The paracellular resistance of co-cultures was above that of RTgutF monolayer, but below that of RTgutGC monolayer (Fig. 3b). This result is explainable by the likely mixture of the two cell lines, resulting in non-continuous tight junction formation between epithelial cells and fibroblasts. Thus, the formation of a natural basement membrane between the two cell types, as demonstrated in a co-culture model of primary rat intestinal endodermal cells and fibroblasts (Simon-Assmann et al. 2007), is unlikely and makes the physical separation of RTgutGC and RTgutF, e.g. through a permeable membrane, necessary.

Following the transcellular resistance profile during monolayer formation (Fig. 3c) it was found that RTgutF and RTgutGC exhibit almost identical resistance values with a steady increase over the culture period. The increase correlated with the doubling time of 4–5 days for both cell lines. Further, the transcellular resistance of co-cultured cells (Fig. 3d), which comprises two cell layers, is almost double compared to the confluent monolayers of RTgutF and RTgutGC. For all three approaches, transcellular resistance values remained stable between day 1 till day 7 of culture, reflecting the stagnant or slow cell growth of high density cultures. Thus, the transcellular resistance is not only capable to inform about cell viability and cell death as shown by Meissner et al. (2011), but also about cell proliferation and cell density. Notwithstanding, the decline of transcellular resistance for co-cultures at day 10 may indicate the start of a critical shortage of nutrients accompanied with decreasing cell viability due to nutrient undersupply of the lower cell layer, which may arise from the continuous proliferation of the two cell lines.

The comparison of electrical properties of RTgutF and RTgutGC provided further evidence of the fibroblast nature of RTgutF. Co-culture initiation on solid support was not beneficial for the overall resistance of the epithelial-fibroblast cell arrangement, which was used as quality control for a functional co-culture model. Therefore, the next step comprised the culture of epithelial cells and fibroblasts on permeable membrane supports.

### Reconstruction of the intestinal barrier on ultrathin, porous membranes

Previous established ultrathin and highly permeable alumina membranes (Drieschner et al. 2017) were used as artificial basement membrane analogue to support co-culture of epithelial RTgutGC and fibroblastic RTgutF cells in a physiologically realistic manner.

Cells were either cultured as monolayer or in two distinct configurations, with RTgutF and RTgutGC in direct contact (co-culture contact) or separated via the alumina membrane (co-culture separate). Transepithelial electrical resistance (TEER) analysis was used to determine the effect on barrier tightness (Fig. 4a). RTgutF monocultures developed the lowest resistance with ~ 13 Ω*cm^2^ during the whole culture period. RTgutGC and co-cultures with RTgutGC seeded on top of RTgutF (contact) exhibited slightly higher resistance values of 15–18 Ω*cm^2^, which remained stable from day 1 to day 10 of culture. These values are equivalent with previously reported resistance values of RTgutGC monolayer cultured on alumina membranes (Drieschner et al. 2017). Only when RTgutGC and RTgutF cells were cultured on opposite sides of the membrane (separate) a positive effect on barrier tightness with an almost two-fold increase in TEER, compared to RTgutGC monolayer, was observed. This supports two ideas: (1) physical separation of epithelial cells and fibroblasts in vitro could be considered beneficial for maintaining the functional architecture of the intestine as found in vivo; (2) the potential cellular cross-talk at the epithelial–mesenchymal interface may improve barrier function by enhancing barrier tightness. The role of the permeable support, to act as artificial basement membrane analogue, is clearly fundamental for the success of this model. Ultrathin alumina membranes potentially support fast communication of different cell types through the straight and abundant nanopores and thus may act as superior interface for co-culture initiation. In addition, these membranes allow for sensitive evaluation of TEER, even in the lower resistance range with changes of a few Ω, which is not possible with conventional cell culture membranes (Drieschner et al. 2017).

For further investigation of cellular morphology of separate co-cultures of RTgutF and RTgutGC cells, a transmission electron microscopy image of a cross-section of 10 day old cultures was prepared (Fig. 4b). The image demonstrates cellular monolayer on both sides of the highly porous membrane. Moreover, cells exhibit a flattened morphology, with a cell height of ~ 5 µm for RTgutGC and ~ 3 µm for RTgutF. The difference in cell height among the two cell lines, even when being small, is an additional hint for their origin from different tissues. Fibroblasts are typically thin and elongated in shape (Ossum et al. 2004), while absorptive epithelial cells, lining the intestinal lumen, have a columnar shape with a height of up to ~ 30 µm (Merrifield et al. 2009). The flattened shape of RTgutGC cells, however, is a typical adaptation of in vitro cell cultures, because cells lack important physiological stimulation, such as mechanical forces from fluid flow occurring on the apical surface of epithelial intestinal cells (Kim et al. 2012).

The developed co-culture model of intestinal epithelial cells and fibroblasts on ultrathin alumina membranes opens new possibilities to study the physiological function of the fish intestine in vitro. One interesting application of this model is the investigation of immunological defence mechanisms of the fish intestine because fibroblasts represent an immune competent cell type (Sorrell and Caplan 2009). Further refinement of the model could be achieved by exposing epithelial RTgutGC cells to realistic flow conditions by implementing ultrathin membrane chips into a microfluidic bioreactor. Eventually, the novel barrier model of the fish intestine is attractive for its physiological realism and opens new doors for fundamental piscine intestinal research.

## Conclusion

In conclusion, we established the first intestinal fibroblast cell line from rainbow trout to initiate research on the role of epithelial–mesenchymal interaction in the fish intestine. It is important to note that cell lines offer plethora of biological research material on an economic and ethical justifiable basis and thus help to overcome the current research restrictions on the fish intestine due to its limited access. With the extension of the current epithelial barrier model to a deeper layer of the intestinal wall—the connective tissue, we found a positive effect on barrier tightness when individual cell types were separated by newly developed ultrathin alumina membranes. This may indicate the importance of fibroblasts in acting as modulators of epithelial barrier function by e.g. producing and releasing extracellular matrix proteins and growth hormones. Further, it demonstrates the great potential of microtechnological innovations for the rearrangement of the cellular microenvironment in vitro, which plays a central role for the recreation of true organ analogues that are capable to offer reliable insights in physiological functions. Thus, the newly developed intestinal barrier model, accommodating epithelial and mesenchymal cells, is a first approach to mimic fish gut complexity and further narrow the gap between in vitro and in vivo models.

## Electronic supplementary material

Below is the link to the electronic supplementary material.

Supplementary material 1 (PDF 233 kb)
