# Supplementary material for: Improving a fish intestinal barrier model by combining two rainbow trout cell lines: epithelial RTgutGC and fibroblastic RTgutF
Source: Cytotechnology. 2019 Jun 29;71(4):835–48. doi: 10.1007/s10616-019-00327-0 (PMC6663964; doi:10.1007/s10616-019-00327-0)
Supplement: Supplementary file 1 — Supplementary material 1 (PDF 233 kb) [file 10616_2019_327_MOESM1_ESM.pdf]

Improving a fish intestinal barrier model by combining two rainbow trout cell lines: epithelial RTgutGC and fibroblastic RTgutF

Authors and affiliations

*Carolin Drieschner[1, 2], Nguyen TK Vo[3], Hannah Schug[1], Michael Burkard[1], Niels C Bols[4], Philippe Renaud[2], Kristin Schirmer[1, 5, 6, *]*

1. Department of Environmental Toxicology, Eawag (Swiss Federal Institute of Aquatic Science and Technology), Dübendorf, Switzerland
2. Microsystems Laboratory 4, School of Architecture, EPFL (École Polytechnique Fédérale de Lausanne), Lausanne, Switzerland
3. Department of Biology, McMaster University, Hamilton, Ontario, Canada
4. Department of Biology, University of Waterloo, Waterloo, Ontario, Canada
5. Department of Civil and Environmental Engineering, School of Architecture, EPFL (École Polytechnique Fédérale de Lausanne), Lausanne, Switzerland
6. Department of Environmental Systems Science, ETHZ (Swiss Federal Institute of Technology in Zurich), Zürich, Switzerland

* Corresponding author: e-mail: kristin.schirmer@eawag.ch, phone: +41 58 765 5266

List of content

Figure S 1. Morphology of RTgutF cells at passage 3.
Figure S 2. Relative telomerase activity of three fish- and one mammalian cell line.
Figure S 3. Negative control of Collagen 1A1 and Vimentin staining.
Figure S 4. Reference sequence of COX1 fragment from *O. mykiss*.
Figure S 5. Expression of tight junction protein ZO-1 in RTgutF and RTgutGC cells.

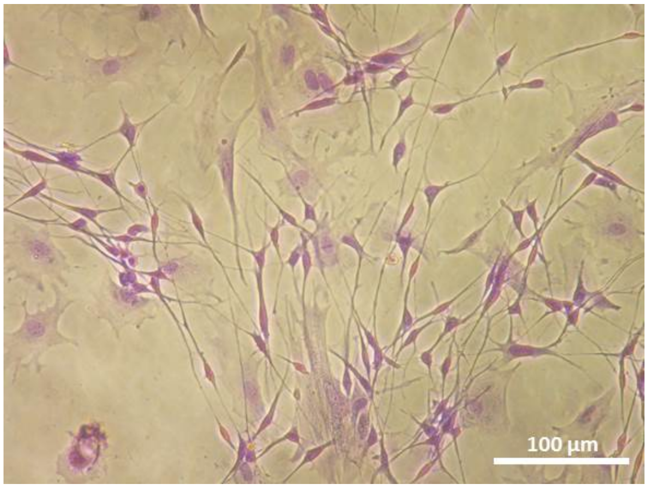

Figure S 1. Morphology of RTgutF cells at passage 3.

Cells were stained with May-Grunward/Giemsa.

Method applied: May-Grunward/Giemsa Staining

Cells were stained with May-Grunward/Giemsa to help visualize their organization and shapes in culture as previously demonstrated by Bloch *et al.* (2016).

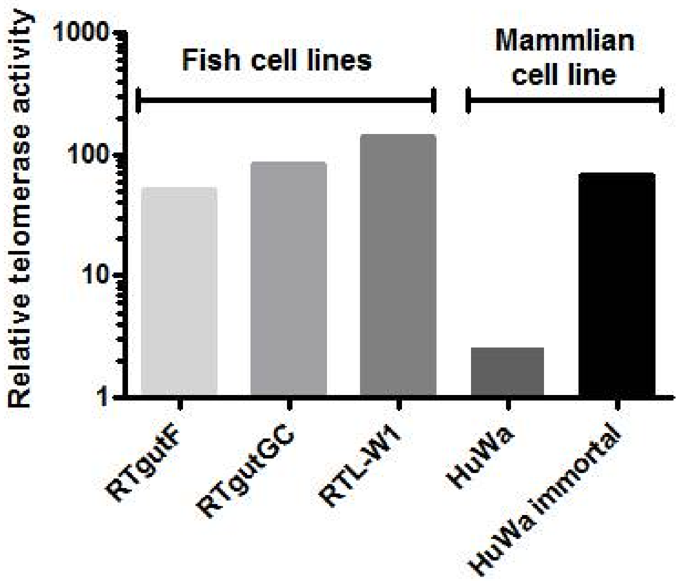

Figure S 2 Relative telomerase activity of three fish- and one mammalian cell line.

Fish cell lines were established from rainbow trout and include RTgutF (intestinal fibroblasts), RTgutGC (intestinal epithelial cells) and RTL-W1 (liver cells). The mammalian cell line HuWa was analyzed before and after immortalization with TERT.

Method applied: Telomerase activity

Telomerase activity was assessed as described for RTgutF in material and methods.

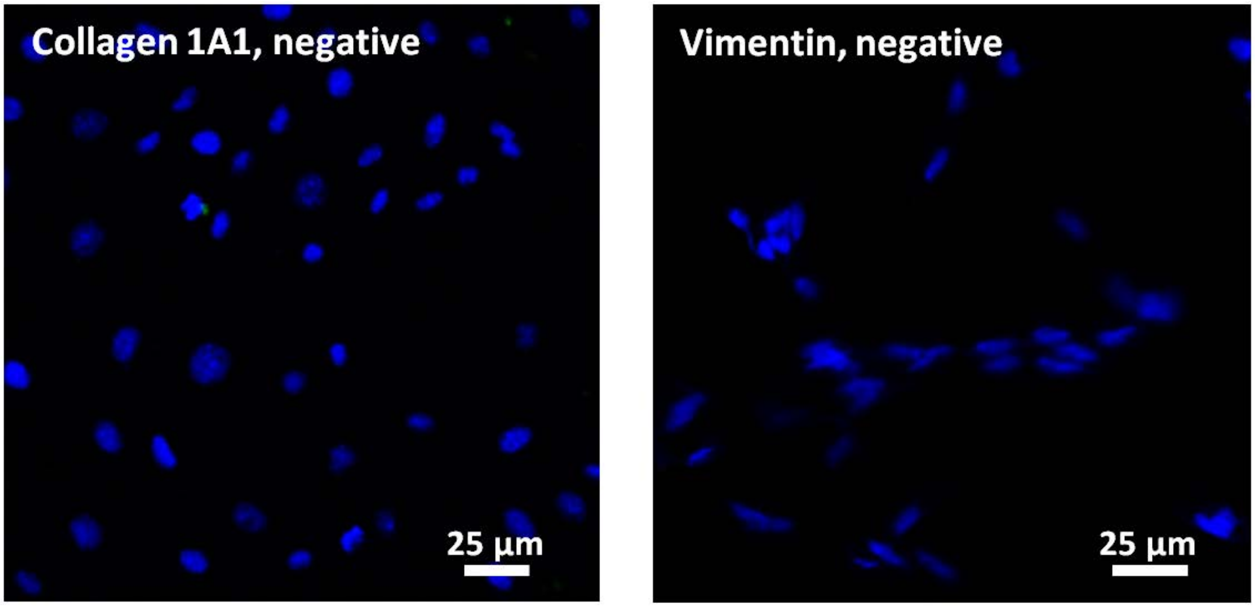

Figure S 3 Negative control of Collagen 1A1 and Vimentin staining.

Cell nuclei of RTgutF are stained in blue. No secondary staining (green) was detected for Collagen 1A1 or Vimentin.

Method applied: Fluorescence Microscopy

RTgutF cells were cultured and stained as described in Material and Methods (Fluorescence microscopy); however, no primary antibody for Collagen 1A1 or Vimentin was applied.

CCTCTATTTAGTATTTGGTGCCTGAGCCGGGATAGTAGGCACCGCCCTGAGTCTACTGATTCGG
GCGGAACTAAGCCAGCCGGGCGCTCTTCTGGGGGATGACCAAATCTATAACGTGATCGTCACA
GCCCATGCCTTCGTTATGATTTTCTTTATAGTCATGCCAATTATAATCGGGGGCTTTGGAAACT
GATTAATTCCCCTAATAATCGGAGCCCCTGATATGGCATTCCCTCGAATAAATAACATAAGCTT
CTGACTCCTTCCTCCATCCTTTCTCCTCCTCCTGTCTTCATCAGGAGTTGAAGCCGGCGCGGGTA
CTGGATGAACAGTATACCCCCCTCTAGCCGGCAACCTCGCCCACGCAGGAGCCTCTGTTGATTT
AACTATCTTCTCCCTTCATTTAGCTGGAATCTCCTCAATTTTAGGAGCCATTAATTTTATTACGA
CCATTATTAACATAAAACCTCCAGCCATCTCTCAGTACCAAACCCCCCTTTTCGTTTGAGCCGTG
CTAGTTACTGCTGTCCTTCTATTACTTTCCCTCCCCGTCCTGGCAGCAGGCATTACTATGTTACT
TACAGACCGAAATCTAAACACCACTTTCTTTGACCCGGCAGGCGGGGGAGATCCAATTTTATAC
CAACACCTC

Figure S 4 Reference sequence of COX1 fragment from *O. mykiss*.

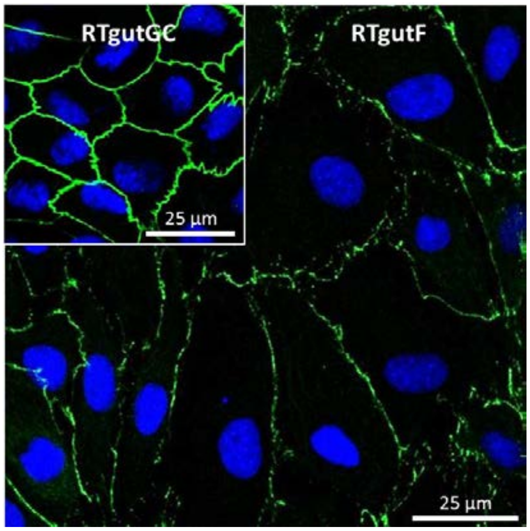

Figure S 5 Expression of tight junction protein ZO-1 in RTgutF and RTgutGC cells.

ZO-1 is stained in green and nuclei in blue.

For RTgutF cells ZO-1 staining patterns reveal a weak and discontinuous line on the cell-to-cell boundary, while RTgutGC cells form a strong and continuous line at the cell boarders.

Method applied: Fluorescence Microscopy

RTgutF and RTgutGC cells were cultured on coverslips (Thermanox, Thermo Fisher, Switzerland) and stained as described in Drieschner *et al.* (2017).

References

Bloch, S. R., Vo, N. T., Walsh, S. K., Chen, C., Lee, L. E., Hodson, P. V. and Bols, N. C. (2016) 'Development of a cell line from the American eel brain expressing endothelial cell properties', *In Vitro Cellular & Developmental Biology-Animal*, 52(4), 395-409.

Drieschner, C., Minghetti, M., Wu, S., Renaud, P. and Schirmer, K. (2017) 'Ultrathin Alumina Membranes as Scaffold for Epithelial Cell Culture from the Intestine of Rainbow Trout', *ACS Appl Mater Interfaces*, 9(11), 9496-9505.
